# The mediation of social influences on smoking cessation and awareness of the early signs of lung cancer

**DOI:** 10.1186/1471-2458-14-1043

**Published:** 2014-10-07

**Authors:** John Chatwin, Andrew Povey, Anne Kennedy, Tim Frank, Adam Firth, Richard Booton, Phil Barber, Caroline Sanders

**Affiliations:** School of Nursing, Midwifery, Social Work & Social Sciences, University of Salford UK, Allerton Building, Salford, M6 6PU UK; Centre for Occupational and Environmental Health, University of Manchester UK, Manchester, UK; Faculty of Health Sciences, University of Southampton UK, Southampton, UK; General Practice Research Unit, University of Manchester UK, Manchester, UK; Wythenshawe Hospital, Greater Manchester UK, Manchester, UK; Centre for Primary Care, University of Manchester UK, Manchester, UK

## Abstract

**Background:**

Whilst there has been no clear consensus on the potential for earlier diagnosis of lung cancer, recent research has suggested that the time between symptom onset and consultation can be long enough to plausibly affect prognosis. In this article, we present findings from a qualitative study involving in-depth interviews with patients who had been diagnosed with lung cancer (n = 11), and people who were at heightened risk of developing the disease (n = 14).

**Methods:**

A grounded theory methodology was drawn upon to conduct thematic and narrative based approaches to analysis.

**Results:**

The paper focuses on three main themes which emerged from the study: i) fatalism and resignation in pathways to help-seeking and the process of diagnosis; ii) Awareness of smoking risk and response to cessation information and advice. iii) The role of social and other networks on help-seeking. Key findings included: poor awareness among participants of the symptoms of lung cancer; ambivalence about the dangers of smoking; the perception of lung cancer as part of a homogenisation of multiple illnesses; close social networks as a key trigger in help-seeking.

**Conclusions:**

We suggest that future smoking cessation and lung cancer awareness campaigns could usefully capitalise on the influence of close social networks, and would benefit from taking a ‘softer’ approach.

## Background

The possible influence of delays in diagnosis on survival and the risk factors for delay in patients with cancer have been the subject of considerable interest and controversy for many years [1]. Survival from cancer in the United Kingdom is poorer than that of other European countries, and it has been suggested that this can be attributed to a more advanced disease stage at presentation [2]. Cancers in the UK are diagnosed later and at more advanced stages than in other European countries with resulting low national survival rates [3]. The situation is particularly serious in the case of lung cancer, where for up to 80% of patients, their disease is found to be inoperable because it has been diagnosed too late [4]. In the UK, it appears that the picture is particularly bleak, with delays in the diagnosis of cancer being attributed to both patient and healthcare system factors [5]. Corner *et al.* [6] reported that delays of up to a year following the onset of worrying symptoms were not uncommon before patients decided to seek medical help. This is in contrast to much of Europe where delays can be far shorter, and survival rates correspondingly higher [7, 8]. In the USA too, it has been found that delays in seeking treatment can be shorter than in the UK, with Jensen *et al.* [9] for example, reporting that such delays can be less than six months. It is further evident that long-term smokers, particularly those with other conditions such as Chronic Obstructive Pulmonary Disease (COPD), and those living alone are at a higher risk of taking longer to consult with symptoms of lung cancer [10]. The reasons for this are a complex mix of individual and psychosocial factors [11, 12], but it has been established that one recurring feature in the case of lung cancer is that patients, regardless of how well progressed their disease was, failed to recognise the seriousness of their symptoms, and reported that they had noticed changes in their health status long before they sought medical help [13].

As yet there is no clear consensus on the potential for earlier diagnosis of lung cancer [14, 15], although research has suggested that the time between symptom onset and consultation can often be long enough to plausibly affect prognosis [10]. Moody *et al*. 2004 [16], for example, highlight that the most effective curative option for lung cancer is surgery, and although this modality has not changed significantly for many years [17], it can be highly effective for patients with early stage disease. It is far less so for those whose disease is more advanced. So it follows that a significant impact on survival rates could be achieved if a higher proportion of patients were diagnosed early enough to benefit from potentially curative surgery.

Current government initiatives in the UK and other countries, aimed at raising awareness of the early symptoms of lung cancer illustrate the importance being placed on this issue at a policy level. The 2005 guidance from the National Institute for Health and Clinical Excellence (NICE) on the diagnosis and treatment of lung cancer, for example, were recently updated to advise healthcare professionals of important advances in management and patient information strategies [18]. And research into public awareness of cancers in the UK has also lately been stimulated by the strategic input of the National Awareness and Early Diagnosis Initiative (NAEDI) [19]. This initiative, launched in 2008 aimed to involve public and third sector organisations to support activities that promote the early diagnosis of cancer and has been instrumental in establishing a number of local and national cancer awareness projects [20].

In this paper, we present an analysis of interview data collected as part of the *Lung Symptom Interpretation and Diagnosis Study* (LUSID).^1^ The study aimed to investigate the social factors which influence symptom recognition and help-seeking behaviour, and focused on two distinct groups:1) patients with an existing diagnosis of lung cancer; 2) those at heightened risk of developing the disease. These groups were chosen in order to understand how perceptions and action in relation to help-seeking might change and develop across the illness trajectory.

## Method

This was a qualitative study which utilised the principles of grounded theory [21] and aimed to systematically interleave data collection, analysis, constant comparison and theorizing in an attempt to produce a plausible representation of the world view of participants that was rooted in their accounts, rather than being hypothesis driven. We also drew on existing material that emerged as relevant to our understanding as the analytical process developed.

Data were collected in 2012 and 2013, and we were able to included 25 participants from two groups; people with an existing diagnosis of lung cancer (n = 11); and people at a heightened risk of developing lung cancer (n = 14), i.e. long term smokers (that is, people who have smoked for over a year), and people with Chronic Obstructive Pulmonary Disease (COPD). The study was situated in two clinical settings in the North West of England. One was a dedicated specialist lung clinic in a large regional hospital, and the other was a local GP practice. All of the diagnosed participants were recruited from the lung clinic along with 3 ‘at heightened risk’. The remaining ‘at heightened risk’ patients came directly via their GP, or from responding to recruitment posters at the GPs clinic. Both the clinic and the GP practice were situated in an area of high economic deprivation on the outskirts of a major (UK) Northern industrial city, and a large proportion of patients in both settings lived locally. The mean age of the diagnosed group was 69 years, and 64 years for those ‘at heightened risk’. In the lung clinic setting, 3 patients who were approached by their consultant declined to take part; 4 ‘at heightened risk’ patients from the GP surgery declined. In all cases initial contact was made via a health care professional (either the person’s GP or their consultant) who obtained provisional consent, and passed their contact details on to the research team. A researcher then arranged to conduct a 30-60 minute semi-structured interview where written consent to participate was obtained. Participants were able to have a relative or carer present during their interview, and 4 participants took up this option. 6 participants who gave verbal consent to be contacted, subsequently chose not to go ahead with an interview and were not included in the study (4 from the ‘at risk’ group, and 2 who had been diagnosed). Resources permitted a fieldwork period of six months, after which time we closed recruitment. The study adheres to the RATS guidelines for reporting qualitative studies.

Interview guides for the in-depth interviews were generated from themes emerging from an initial literature review [22], formal and informal contacts with key informants, and input from PPI (Public and Patient Involvement) representatives. This comprised of formal consultations with an established PPI network at the University of Manchester (the PRIMER group).^2^ PRIMER were able to provide input from an early stage of the project. Two representatives from this PPI group also joined the project steering committee and contributed to aspects of the analysis (see Acknowledgements). Ethical approval for the study was obtained from the regional NHS ethics committee (REC reference 12/NW/0592).

### Analysis

The main topics included in the interview schedule covered participants’: *current understanding of the early signs and symptoms of lung cancer*; *perceptions of the seriousness of symptoms; how relevant health related knowledge was obtained; the role of social networks; triggers to seeking treatment.* The schedule was used flexibly however, and developed as new lines of inquiry emerged. Interviews were recorded and transcribed verbatim; transcripts were anonymised.

Analysis followed the principles of grounded theory [21]. Transcripts were examined using a process of focused coding, categorisation and thematic development. Following an initial analysis, emergent themes were discussed within the project team and with PPI representatives in an ongoing process which enabled potential early findings to inform subsequent interviews. This refinement and incorporation continued throughout the fieldwork period. Meanings drawn from relevant field notes and interpretations arising during the process of the research were also incorporated where resonant with the data.

## Results

Three main themes emerged which encompassed key elements of participant experience in the context of this clinical arena, these were: *i) Fatalism and resignation towards pathways to help-seeking and the process of diagnosis. ii) Awareness of smoking risk and response to cessation information and advice. iii) The role of social and other networks on help-seeking.*

### i) Fatalism and resignation towards pathways to help-seeking and the process of diagnosis

A strong thread with our diagnosed participants was a kind of fatalistic attitude towards post-diagnosis processes. Cancer fatalism - the belief that death is inevitable when cancer is present - has often been identified as a barrier to participation in cancer screening, detection, and treatment [23]. However, in this pure sense, it was not particularly evident in our data. Elements of fatalism, where they were apparent, were more akin to resignation about having to go through a potentially painful and ultimately tenuous (in terms of possible outcomes) treatment process. Participants essentially saw their treatment as external – something basically unpleasant that needed to be done *to* them, rather than something they had any real agency over.

Participants were happy to ‘leave it to the doctor’ and there were few instances where diagnosis appeared to lead to more than a limited interest in the mechanics of their illness, beyond a basic understanding of the treatment processes that were being recommended. A similar situation existed in relation to potentially worrying symptoms, or ones that might indicate the early stages of lung cancer. When asked about symptoms that might have been regarded as serious enough to seek medical help, for example, this participant - who was shortly to undergo surgery on his lung -commented:

Probably as I feel now, you know, I’ve got a lot of tightness in my chest and the occasional pains and that, I would have thought that was relatively serious, but in fact that wasn’t even beginning to happen when I first, you know, went through the process, it’s only in the last couple of weeks I began to feel this, and I’m not even sure that it’s because I’m aware of it. But up to that point I couldn’t say I had any clue that it was anything serious, I just thought it was something that, some pill might get rid of this cough for me. (Participant 01)

Attitudes were similar in the ‘at risk’ group. Particularly in terms of the potential to be resigned or even fatalistic about their prospects should they develop cancer. (In this and the other extracts presented R = ‘respondent’; I = ‘interviewer’.)

R: But even what I’ve got [COPD]; I just think to myself, you know, you’re told you’ve got it. And I thought, well, I’ve got it, you know, they’ve given me medication. I struggle along as I do, and I thought, well, I’ve got it, you know, get on with it. You have to put up with it.

I: So you don’t really want to know more about it?

R: Not really, I’m not very good. I mean, I’ve been very lucky over the years because, I mean, it’s only since the last few years, say about the last five or six years, that with the bowel and this, I started getting all this. And beforehand, I was so healthy, you know, I never had nothing. And I think I’m just one of these. If I’ve got it, I take the medication. There’s days I’m not very good, so I’ll go to the doctor and tell them what it is, they’ll tell me what to do,or change my inhalers and things. I just take it as it comes. (Participant 21)

There was often the admission that looking out for, or acknowledging the appearance of worrying symptoms was something that people chose not to think about in any formalised way. When prompted, participants would usually mention some of the more serious (and not necessarily early) symptoms of lung cancer such as coughing up blood or severe pain. This attitude was also coupled with a reticence about addressing the possibility of cancer, even if symptoms might indicate this. While, if it cropped up in an interview, ‘coughing up blood’ was universally acknowledged as something warranting immediate medical attention, its appearance might be attributed to any number of other lung problems apart from cancer. And, particularly among some of the older respondents - who were likely to be suffering from other age related illnesses - there was a tendency to group various unrelated medical conditions together; they would attribute no special significance to cancer as a particularly serious (i.e. potentially terminal) disease, and make little differentiation between different types of cancer. This 82 year old female participant reported that:

R: I see it as a whole thing, you know the cancer and the lung thing, my legs, I put it all together and just think oh well that’s the way it is.

I: So if you did start coughing up blood or something like that you wouldn’t be thinking ‘cancer’ you’d be thinking, oh it’s more of the same?

R: Yeah if I was coughing blood up I’d send for the doctor.

I: But you wouldn’t be specifically worried that it must be cancer?

R: No I don’t think like that. No. I think I might have burst a blood vessel. (Participant 07)

Information seeking, and the sources of information that people found useful in relation to health matters generally, and lung cancer in particular, were surprisingly limited. The majority of participants from both groups indicated that they made frequent use of computers and the internet. However, this tended to be focused on social activities such as staying in touch with friends, rather than active information seeking. Among the ‘at risk’ group there was little sense that health information websites such as NHS direct were directly influential on knowledge and awareness about lung cancer – or any other serious condition. Diagnosed participants who used the internet generally reported that they had researched their illness post-diagnosis, but their interest remained relatively pragmatic; they might look up their particular type of cancer to understand what the treatment process would involve, but would not necessarily be engaged in searches for alternative treatments or novel cures. There was some evidence however, that close members of respondents social and family networks might engage in this type of activity:

My nephew has the internet, and he looked, I think he was looking at it the other week. He went, good God, [name], he said, this is not like just asthma or anything. And I says, I know, sweet, I got told its one over, you know, it’s crossed the line from asthma. . . And he went, God, [name], it don’t look good on there, how you could end up. And I thought, oh brilliant. (Participant 21)

### ii) Awareness of smoking risk and response to cessation information and advice

Interviewees were specifically asked for views on the current and past (UK) health awareness campaigns – including a recent national (UK) TV campaign that directly focused on watching for the early symptoms of lung cancer, and more general campaigns aimed at emphasising the dangers of smoking. It was clear that smokers particularly disliked the more graphic current TV adverts, and ignored them almost as a matter of principle.

R: what I did was, right, rather than look, I just used to turn over. But if I’d have not turned over and kept watching it, which I should have done, then probably I would have given up earlier.

I: So you think they would have had an impact on you? If you’d have watched?

R: Yeah I do.

I: But you knew that they were going to give you a message you didn’t want, so you’d turn it off?

R: That’s why I turned it off quick, yeah. Yeah. And like when they came on the [cigarette] packets. . . And I looked at what they - it could do - and I thought, oh my God. I used to hide the packet, or put them in a case. (Participant 19)

R: [Referring to graphic anti-smoking campaigns] There’s always things on telly about smoking – giving it up. In the newspapers there’s things about it as well. The cigarette thing [referring to a recent advert] is awful I must admit, but it doesn’t put me off whatsoever. (Participant 16)

Participants tended to be ambivalent about the effects of smoking and, if they were still smoking, the likelihood that they would attempt to give up. On the one hand, participants would readily admit that there was a real risk of lung cancer (or other health implications) if they continued to smoke. But on the other, the reasons they cited for not giving up outweighed this. Several diagnosed participants, for example, reported that they continued to smoke but justified the situation by saying that the stress of giving up would make their lives more difficult; they regarded the damage as having already being done. Cutting down was often seen as an acceptable compromise. All participants who smoked reported having been advised to stop by their doctor. However, as with overly graphic or instructional anti-smoking media campaigns, the way in which this issue was broached in a consultation had a significant impact on whether or not the advice was taken. ‘Softer’ approaches from health care professionals tended to have more impact than overly authoritative or instructional communication styles:

He [the surgeon] was really nice and he was…you know, he said, look, he said, you can smoke, if you want to, but we would prefer it if you didn’t smoke, at least two weeks before you’re due to have your operation, I mean, I had to go and have all the tests to see if I was fit enough to have the operation and then…and I said, right, okay, and, I think, July, I gave up in the July as I had my op in the September, or I might have had the odd puff on a cigarette, maybe two drags of a cigarette if I’m honest.” (Participant 22)

[Commenting on a doctor who forcefully told her to stop smoking] It makes you feel worse, rather than better. You know, I like it- if I go to a doctor – and he’ll say, well there’s no good point me telling you to stop smoking, you’ve been smoking all your life.

It’s up to you. That is better than a doctor coming right out and saying stop smoking now.

No way. I mean I was told to stop when I had my stroke. They said I’d be dead within 10 years if I didn’t stop smoking. . . oh he [the doctor] was awful. He was nasty. I couldn’t believe a doctor was like that. It was because I was in [nurses] uniform and I smoked. And I was furious with him and I said I will not stop smoking, nobody tells me what to do. (Participant 16)

Most participants said they’d go to their doctor if they started noticing persistent symptoms such as coughing, or ‘a different kind of coughing’. However, as outlined already, these wouldn’t necessarily be attributed to cancer, or the possibility that these could be early symptoms. There was little reference to the potential for lung cancer to have a trajectory of symptoms or signs, and hardly any acknowledgement that recent awareness raising campaigns in the media specifically targeting this issue early might have any influence.

### iii) The role of social networks on help-seeking

It appeared that close family – particularly partners or spouses – had the biggest impact on decisions about when to seek medical help when symptoms of concern became evident. This diagnosed participant was typical in that after exhibiting symptoms for some time, it was his partner who eventually persuaded him to go and see his doctor:

I can’t believe, in fact I was bullied into going by somebody else in the end, and in truth I probably would have been longer before I went. (Participant 01)

Members of extended networks, such as neighbours reportedly had little influence unless they were regarded as close friends, and participants noted that wider and more nebulous networks such as those existing around activity specific clubs or groups were even less important.

I: So can I ask you then, apart from doctors and health professionals, who would you trust to give you advice on your health and seeking advice, if you had symptoms?

R: Well, I don’t…I mean, I think…I have a friend who is a retired GP and I would trust her, but the trouble is, everybody has got their different opinions as to what’s the best thing to do.

I: And their own experience comes into it, doesn’t it, whatever they’ve gone through?

R: Yes. So, no, I would, basically, go with what my GP said really.

I: And your husband?

R: Well, yes, yeah.

I: But the trouble is, everybody has got their different opinions as to what’s the best thing to do. (Participant 03)

(In this extract, W is the respondent’s wife.)

R: Well it’s old wives tales isn’t it? People say oh you don’t want to do that, you want to do this. You don’t want to do this you want to do that. If I was going to take anything seriously I’d take it off a doctor rather than… [gestures towards his wife –both laugh]

W: Oh he doesn’t listen to me. No he doesn’t listen to me.

R: Now and again you start and I say, oh I’ve got a bit of a sore throat. You say, oh get one of these lozenges down you. Things like that. But I mean anything big time you know, I wouldn’t ask anybody in the pub or [anything] like that. (Participant 15)

Friends, neighbours and acquaintances who had suffered cancer, or cared for someone with the illness, however, were particularly valued as sources of informal information and support. Again, it was not necessarily important that these people had experienced the same type of cancer as the participant, but rather that they had had close involvement with serious life threatening illness. This gave them an experiential credibility that others lacked:

I’ve just had a call from the MacMillan nurse who is going to come around and, obviously, I’ve got a huge regard for MacMillan nurses and, I suppose, just talking to people who have had cancer and, you know, I watched my brother-in-law not survive, but watched other people survive and, you know, my own mother battled on with it, it might have been better if she hadn’t survive, because she was in a lot of pain, but she had breast cancer in the late 50’s and survived until she was 80, yes, in her 40’s and survived until she was 80, so anecdotally, well, we all know people, don’t we. (Participant 02)

Gender differences (or more specifically, the reinforcement of gender stereotypes) were relatively undefined, however all of the male participants in the study said they tended to avoid going to the doctor for *any* reason, and were usually pressurised into it by a spouse or family member. The comment by this participant’s wife (W in the transcript) emphasised how extreme these tendencies could be:

I: [To male participant] So you’re the kind of person who just battles through?

R: Yeah.

I: What would make you go to the doctor?

R: What would I go for to the doctor?

I: What would be serious enough for you to want to go to the doctor, if there is anything?

W: If they were screwing the lid down on his coffin!

R: I’d have to be dying! I’d have to be dying, yeah.

I: So you wouldn’t normally.

R: No, no, no. (Participant 06)

Although reluctance to engage with medical services in general was in evidence, it did not appear that actually going to the doctor was the issue. Rather, it was the level or severity of symptoms that would trigger a visit; these were very high in some people to the point where they reported that they would have to be in acute pain or state of debilitation before initiating contact themselves. Again, this links with the finding that spouses and close family members were the primary trigger for many people to seek medical help.

R: In fact I’d go as far as to say if I hadn’t got a girlfriend who had been pushing me it’d have probably only been in the last fortnight I’d have gone to the doctor because I’m beginning to, you know, feel some symptoms now.

I: But even if you’d have gone now you still maybe wouldn’t have connected it with cancer would you?

R: No, I wouldn’t…

I: …you’d have thought chest pains…

R: …not with chest pains no. (Participant 01)

## Discussion

A striking feature of our data was the relatively limited interest that participants appeared to have in the signs and symptoms of lung cancer. This is perhaps understandable for those at heightened risk because even though it was technically accurate to describe them as such, this categorisation does not appear to have been a major feature of how they actually saw themselves on an everyday level. There may have been a degree of avoidance occurring too, so that people appeared reluctant to engage with the issue not because they weren’t interested but because to do so might be felt to be too pessimistic, or tempting fate in some way. That this was also evident in our diagnosed participants was perhaps surprising, but if thought of in terms of avoidance or distancing being used as a conscious or un-conscious coping strategy [24], it becomes more explicable. We did not undertake a specific measure of psychological distress, and so are not able to provide definitive evidence that this was in fact what our diagnosed participants were engaged in. However, a higher level of distancing (which reportedly allows the patient a greater capacity to gradually assimilate their situation, rather than having to deal with it head on) has been associated with lower levels of psychological stress in many forms of cancer [25, 26].

At a practical level, there have been some efforts to investigate the factors which influence how people judge whether or not changes in their health status are serious – and whether or not they decide to visit their doctor. Smith *et al.* [10] for example, undertook a qualitative synthesis of 32 research papers dealing with the help-seeking experiences of patients with 20 different types of cancer (>775 patients and carers). They found that there were strong similarities in patients across all cancer types, and that key concepts were the recognition and interpretation of symptoms, and fear of the consultation. Focusing specifically on lung cancer, Corner *et al.* [6], interviewed 22 patients who had recently been diagnosed with operable (early stage), and inoperable (late stage) lung cancer. They concluded that regardless of their disease stage, or their social background, these individuals failed to recognise symptoms that they had experienced over many months prior to their eventual diagnosis as particularly serious or warranting medical attention. The findings of our study resonate strongly with these two studies, but may further indicate that a tangible and deliberate lack of engagement with potentially serious symptoms (along with a clear underlying knowledge that they may occur) is also at work. The ‘downgrading’ of symptoms and the tendency of people to attribute them to less serious illnesses when they did occur was also evident in our data. This finding is broadly in line with the literature on health related help-seeking behaviour in general [27], but also registers strongly with research more specifically focused on cancer patients. A study by Mor *et al.* [28], for example, provided a content analysis of remarks made by a corpus of 625 lung, breast and colorectal patients, and concluded that patients suffering from lung or colorectal cancer were significantly more likely to blame their symptoms on other less serious illnesses, and thus delay seeking medical help. Mor *et al.* [28] provide no specific data on the nature of the health changes that patients failed to act upon, or chose to downgrade. However later work, including that of Tod *et al.* [4] and Braybrook, [29] is more definite, and outlines symptoms such as persistent or irritating coughs, changes in the weather, and ‘getting older’ as those that patients routinely describe retrospectively after diagnosis. Again, this is resonant with the accounts we collected. Essentially, as Chapple *et al.* [30] suggest, there may be a strong tendency for people to normalise symptoms.

An interesting detail to emerge was the tendency for some participants (particularly those with lower socio-economic status (SES) to bracket together any number of different medical conditions. Breathlessness, joint problems, heart problems and different types of cancer were all viewed as a kind of single homogenous condition. This may reflect the predominance of low health expectations in our sample, and an underlying acceptance of the inevitability of physical decline, but it may also be connected to a degree of ‘cancer fatalism’. This concept has been oporationalized in different ways in the literature, and has attracted a degree of philosophical as well as psychological attention (See, for example, [31, 32]). A number of studies have established a link between people who have a lower SES and higher levels of fatalism ([33–35]). Further, one study of 2018 British adults found that people with low SES were less positive about early cancer detection, and more fearful about seeking help for a suspicious symptom [34]. While a population based survey, also conducted in the UK, reported a link between low SES, lower awareness of cancer warning signs and greater anticipated delay in seeking help [36]. In the context of this study we used the term in a very broad sense to incorporate not only the belief that an inevitable consequence of developing cancer will be death [23], but that various lesser degrees of resignation toward the trajectory of the illness can be observed.

To date there has been a limited body of work focussing directly on social networks and chronic illness, although some research has dealt with aspects of social support [37]. Distinctions have been drawn between the strong social ties which develop through close personal relationships such as those within families, versus weaker ties with civil organizations and other more formal groups. It has been suggested that groups with lower SES tend to depend more on strong ties whilst the middle classes are more adept at making use of weak ties [38, 39]. This did not appear to be the case, however. Although the importance given to wider networks *per se* by participants was broadly in line with studies which have identified the processes of social network engagement, it was really only very close family contacts that were reported to be influential in terms of cancer or other serious illness related issues. This echoes recent research highlighting that the greatest contribution to illness management within personal social networks of those with chronic illnesses, comes from partners and close family members [38]. Age may have been a factor here as well: A recent multi-cancer meta-analysis by Pinquart & Duberstein [40], for example, examined 87 studies on the associations of perceived social support, network size, and marital status, and although levels of engagement varied by cancer type, social network correlations were found to be generally stronger in younger patients. Our sample was predominantly comprised of older individuals, and our findings strongly support several other studies which have highlighted the role that very close family and spouses (as opposed to slightly wider community networks) play in prompting people to seek medical help when they notice potentially serious symptoms. Along with straightforward legitimisation of symptom severity – a theme which crops up in work on a variety of cancers (see, for example, [4, 29]) – such close networks can also perform the function of actually sanctioning a visit to the doctor. This ‘legitimisation of the appointment’ was found to be particularly important to male patients who were concerned that they would not be seen as wasting the doctor’s time with trivial symptoms, but the effect has been noted in some female patients too [10]. The issue of informal network legitimisation is particularly relevant in the context of our study because it suggests that people who have limited engagement with very close social networks may be at a disadvantage.

Early work by Goodwin *et al.* [41] noted significant connections between marital status and diagnostic delay in lung cancer, with those individuals who lived alone showing far more of a tendency to delay help-seeking than those in stable or long term relationships. More recently, Neal and Allgar [42] utilised the secondary analysis of patient-reported data from the ‘National Survey of NHS patients: Cancer’ to explore the relationship between socio-demographic factors and the components of diagnostic delay. And in line with the earlier findings of Goodwin *et al*, Neal & Allgar [42] reported that across six cancers (lung, breast, colorectal, ovarian, prostate, and non-Hodgkin’s lymphoma), it was single and separated/divorced people who had delayed longer than married people in seeking medical help.

Unusually, considering that recent data collated by Cancer Research UK indicates that smoking is directly responsible for 90% of lung cancers [43], a number of participants in our diagnosed group were either not currently smokers, or had never smoked. This gave us the opportunity to explore the issue of smoking related stigma – something which has been widely reported as presenting difficulties for patients who have never smoked [44]. Chapple *et al*. [30], for example, reported that non-smokers felt particularly stigmatised because of the association with smoking that lung cancer can have. In more recent work too, Sun *et al.* [45] describe how non-smokers with cancer often carry the stigma that their cancer is self-induced, and can feel ostracized by the public and abandoned by the oncology community. In our data, however, negative issues such as these were not strongly evident, even among those who had non-smoking related lung cancer. Work by De Nooijer *et al*. [46] has suggested that shame and embarrassment about symptoms actively hinders early presentation and diagnosis, but again, this was not the case in the people we interviewed. A possible reason for this may be linked to the social expectations, demographic makeup and age range in our corpus; none of our participants, for example, were under 57 years of age, and Menec & Perry [47] have suggested that stigma related to lung cancer is more likely to be associated with younger age groups.

The relationship between smoking and lung cancer risk is almost universally acknowledged. In a study based on data supplied by Cancer Research UK, Redeker *et al.* [48] reported a 90% awareness of this association. However, other recent work by Simon *et al.* [49] on the development of a robust cancer awareness measure clearly demonstrated that understanding of the symptoms and risk factors for lung cancer is very poor in the UK, with 38% of their large study sample unable to name a single symptom. There is a growing body of research focusing particularly on the psychological aspects of smoking cessation campaigns. Recent work by Borelli [50], for example, focused on the use of motivational interviews by healthcare practitioners at the individual and group level, but acknowledge that decreasing the prevalence of smoking will take multi-target, multi-channel and multi-method approaches. Armitage & Arden [51] were also concerned with the effects of motivational and volitional processes. In a study involving 350 smokers, they randomising participants to one of three groups who received either i) a smoking awareness questionnaire; ii) the questionnaire plus instruction to plan to quit; or iii) a questionnaire, plan to quit and help with forming an implementation plan. In line with the multi-method argument, Armitage & Arden proposed that harnessing both motivational and volitional processes would enhance the effectiveness of smoking cessation programmes, and they in fact found that considerably more people in the third group stopped smoking than in the first. Significantly too, individuals who were already motivated to stop smoking at the point of entering the study had most success in giving up. In a similar vein, recent qualitative work by Park *et al.* [52] concluded that although smokers often perceived smoking as a high-risk activity for lung cancer and smoking related diseases, this heightened concern in its self rarely motivated individuals to seek screening.

These findings are in line with reports from our participants over what would most influence them to stop smoking – the consensus from smokers in both groups being that individuals need to be self-motivated to stop smoking (for whatever reason), and that without this basic level of intention, any health promotion interventions are likely to be unsuccessful. The almost universal rejection of anti-smoking campaigns which are becoming ever more graphic and hard hitting, is an issue that also needs to be addressed if ‘hardened’ smokers are not to become alienated and unreachable.

The ambivalent attitude towards the dangers of smoking that we found in both our diagnosed and ‘at heightened risk’ groups, and a tendency for people to take a relatively perfunctory attitude towards the potential symptoms of lung cancer (or, if they were diagnosed, the details of their illness) may have been influenced by the particular social context of our corpus. Molassiotis *et al*. 2010 [5] outlined how certain socio-demographic, psychosocial and clinical characteristics can have an influence on the point at which a person first becomes aware of significant symptoms. In a study involving 75 patients diagnosed with a range of different cancer types, they found that older age, negative beliefs about cancer, fears about the consequences of having cancer, and reluctance to engage with the process of receiving bad news all came into play. Many of our participants were drawn from a relatively poor working class area, and in contrast to more affluent areas, smoking still carries far less of a general stigma than among some other groups [53]. These findings tend to support other work which has focused on the influence of social background and attitudes towards smoking and lung cancer. In particular, comparable trends have been reported by [6, 10, 49], while recent work by Beeken *et al.* [34] found that – partly because of a greater tendency towards fatalism over cancer that we outlined earlier in this article –people with lower socio-economic status saw it as less worthwhile to detect it and seek help early.

### Limitations

Two main limitations of this study should be acknowledged. The first concerns the sample size. While for an in-depth qualitative study of this nature, the data corpus can be considered to be entirely adequate, future studies would perhaps benefit from a broader range of demographic representation. The study population was largely drawn from a fairly deprived inner-city area, so the inclusion of more participants with a higher SES would be useful in unpacking some of the social and generational aspects of the data. Similarly more participants from younger age groups would broaden the study. Another limitation relates to the particular makeup of the ‘diagnosed’ group. Due to the requirements of the recruitment process – which involved potential participants being approached by a consultant during an appointment were they may have just received the news that they had lung cancer – it is likely that our sample is made up largely of people who have a particular type of outlook. The nature of the questions we were concerned with made this particular bias unlikely to influenced our findings, however, in future work, it would be useful to find a way of recruiting people with lung cancer by utilising different types of health networks. The problem will always be, however, that the aggressive nature of lung cancer gives researchers a relatively short window of opportunity between diagnosis and the onset of treatment.

## Conclusions

Against the backdrop of what is a significant issue in terms of health promotion, there is now a strong policy emphasis in the UK not only on helping people to give up smoking, but also on raising awareness of the early signs and symptoms of lung cancer. In this article we have outlined three main themes that emerged from the *Lung Symptom Interpretation and Diagnosis Study*.^1^ These were: *i) Fatalism and resignation in pathways to help-seeking and the process of diagnosis. ii) Awareness of smoking risk and response to cessation information and advice. iii) The role of social and other networks on help-seeking.* We have tried to show that while our analysis confirms the findings of several other studies (notably those concerning the key role of close social networks, and the tendency of people to play down or attribute symptoms to other, less serious conditions), there are areas where our results indicate some divergence from extant work. In particular, we have described the phenomena whereby some respondents homogenised groups of unrelated illnesses, and the way in which this reflects a particular type of fatalistic perspective.

In terms of how our findings might inform future developments in health promotion and information provision in this area, we would suggest that a fruitful avenue to pursue is one that is able to utilise the strong influence of the close social networks we have described. Many of the high impact and high shock value campaigns that continue to be commissioned appear to have limited impact on certain key groups – particularly people who are hardened smokers. Campaigns that attempt to trigger help-seeking or smoking cessation behaviour by proxy in people close to those at heightened risk, rather than targeting the individual themselves may be more effective. Similarly, taking a ‘softer’ approach which does not immediately alienate users may be a better way to engage some groups. As our study has indicated, the present cycle of ever more graphic campaigns often cause those who may essentially be utilising mechanisms of denial or distancing (in the broadest sense) to ‘switch off’. Similarly, we would suggest that – particularly among lower SES communities where smoking is still extremely prevalent – it may be counter-productive to rely on the effect of smoking stigmatisation which has become a feature of more affluent sectors of society. There does appear to be potential to engage with people on a more conciliatory level, although it should be noted that early evaluations on the effect of the most recent ‘non-threatening’ or ‘soft’ national media campaigns linking smoking cessation and lung cancer awareness are at best, inconclusive (see, for example, Durkin *et al.* 2012) [54]. At root, it may be that, as Sweeny *et al.* [55] point out, although acquiring information can provide numerous benefits, people often opt to avoid engaging with it.
